# CRISPR imaging reveals chromatin fluctuation at the centromere region related to cellular senescence

**DOI:** 10.1038/s41598-023-41770-6

**Published:** 2023-09-05

**Authors:** Hideaki Takata, Yumena Masuda, Nobuko Ohmido

**Affiliations:** 1 Biomedical Research Institute, National Institute of Advanced Industrial Science and Technology (AIST), Ikeda, Osaka, 563-8577 Japan; 2 Graduate School of Human Development and Environment, Kobe University, Nada-ku, Kobe, 657-8501 Japan

**Keywords:** Senescence, Centromeres, Cellular imaging, Nuclear organization

## Abstract

The human genome is spatially and temporally organized in the nucleus as chromatin, and the dynamic structure of chromatin is closely related to genome functions. Cellular senescence characterized by an irreversible arrest of proliferation is accompanied by chromatin reorganisation in the nucleus during senescence. However, chromatin dynamics in chromatin reorganisation is poorly understood. Here, we report chromatin dynamics at the centromere region during senescence in cultured human cell lines using live imaging based on the clustered regularly interspaced short palindromic repeat/dCas9 system. The repetitive sequence at the centromere region, alpha-satellite DNA, was predominantly detected on chromosomes 1, 12, and 19. Centromeric chromatin formed irregular-shaped domains with high fluctuation in cells undergoing 5′-aza-2′-deoxycytidine-induced senescence. Our findings suggest that the increased fluctuation of the chromatin structure facilitates centromere disorganisation during cellular senescence.

## Introduction

DNA is organized as chromatin with histone and nonhistone proteins in eukaryotic nuclei. Chromatin has a highly dynamic structure, and its three-dimensional (3D) structure regulates gene expression, DNA replication, and repair^1–5^. The structure is epigenetically regulated; therefore, changes in DNA and histone modifications are associated with cellular differentiation, diseases, and aging^6–8^. Techniques to obtain information on the 3D chromatin structure are essential to determine these epigenetic functions. Hi-C analysis indicates chromatin interaction sites in the entire genome through DNA sequencing after ligating closely located chromatin fibres^9^. Fluorescence in situ hybridisation (FISH) has also been used to visualize 3D chromatin architectures in the nucleus^10^. These techniques have revealed multilevel hierarchical chromatin structures in interphase cells, including chromosome territories, compartments, topologically associating domains and chromatin loops, and some functional aspects of these structures have been determined^11,12^. However, Hi-C and FISH analyses only show a snapshot of the chromatin structure in fixed cells at specific time points.

To study chromatin structure dynamics, the clustered regularly interspaced short palindromic repeat (CRISPR)/Cas9 system, initially developed for genome editing, has been used to label specific genome sequences in living cells^13^. Cas9 binding to DNA is defined by a single guide RNA (sgRNA) that introduces fluorescent molecules to specific DNA sequences using nuclease-deficient Cas9 (dCas9). dCas9 expression with sgRNA can help visualize repetitive sequences, including telomeres, centromeres, and non-repetitive gene loci in living cells, and the dynamics of these sequences can be tracked^13–16^. DNA can be labelled using fluorescent proteins fused to dCas9 or RNA-binding proteins that recognize the RNA aptamer conjugated to sgRNA. Therefore, CRISPR imaging technology is a powerful tool for understanding cellular events associated with 3D chromatin organisation in the nucleus, including transcription and DNA repair^17–20^.

Cellular senescence is an irreversible arrest of cell proliferation and is associated with changes in chromatin organisation caused by cellular stresses such as telomere shortening, irradiation, and oncogenic or oxidative stress^21^. Centromeric disorganisation is a prominent feature of cellular senescence. The centromere is a constricted chromosomal locus required for proper chromosome segregation during cell division. Human centromeres comprise approximately 171-bp long AT-rich α-satellite DNA repeats, and centromere protein A (CENP-A) is incorporated into the α-satellite DNA to form a centromere-specific nucleosome^22^. CENP-A expression decreases in senescent cells^23,24^, and expansion of the centromere region containing α-satellite DNA is detected using Hi-C analysis and microscopic imaging^25–27^. This change in the centromere structure is known as senescence-associated distension of satellites (SADS)^27,28^. However, little is known about SADS due to limited methods for analysing the chromatin dynamics associated with centromere.

Here, we show that CRISPR imaging is a powerful tool to monitor cellular senescence. To detect the centromere structure, α-satellite DNA was visualized using CRISPR imaging, and the dynamics of α-satellite DNA in living cells revealed the centromere-related disorders in SADS.

## Results

### CRISPR imaging of α-satellite DNA in human cell lines

**Figure 1:**
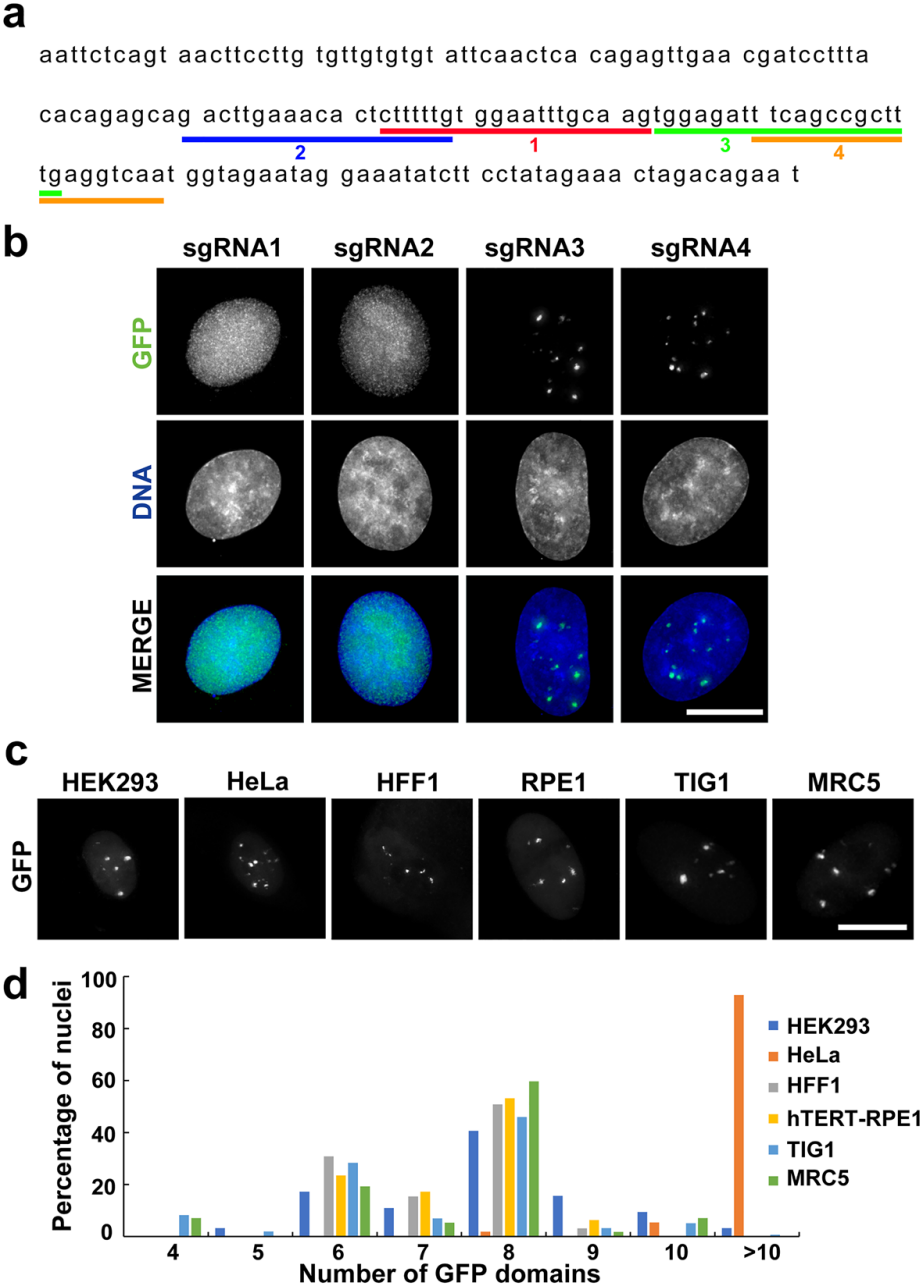
Visualizing α-satellite DNA using CRISPR imaging. (**a**) Targeting sites of the 171-bp α-satellite repeat unit sequence using CRISPR imaging. (**b**) GFP (green) and DNA (blue) signals were detected in HeLa cells transfected with dCas9-GFP- and sgRNA-expressing vectors. Scale bar, 10 μm. (**c**) CRISPR imaging using sgRNA 3 targeting α-satellite in different human cell lines. GFP signals in a single nucleus are indicated. Scale bar, 10 μm. (**d**) The bar graph shows the frequency of the number of GFP domains per nucleus in human cell lines. Over 50 cells were analysed for each cell line.

A centromere repeat sequence, α-satellite, was targeted to observe chromatin dynamics in human cells using CRISPR imaging. The α-satellite is a 171-bp and highly divergent AT-rich repeat, and the monomeric or tandem arrays organise approximately 200 kb–4 Mb of centromere chromatin^29,30^. Green fluorescence protein (GFP)-fused dCas9 (dCas9-GFP) was bound to the α-satellite DNA using sgRNAs designed based on the 171-bp consensus sequence^31^. Several reports on detecting α-satellite DNA using CRISPR imaging exist^32–34^; however, the interference between neighbouring centromere regions might make it difficult to track the dynamic structural changes in a single centromere region. To detect fewer centromere regions, four sgRNAs (sgRNAs 1–4) targeting the α-satellite repeat were designed from an existing α-satellite DNA sequence in the human genome, and not the consensus sequence constructed from the most frequent residues at each site^31^ using the CRISPR design web tool (http://crispr.mit.edu/) (Fig. 1a). The sgRNAs were transfected into HeLa cells with a dCas9-GFP expression vector. sgRNAs 1 and 2 did not show GFP accumulation at specific sites; however, sgRNAs 3 and 4 led to several GFP foci forming in the nucleus (Fig. 1b). sgRNAs 3 and 4 showed similar GFP localisation patterns; therefore, sgRNA 3 was used in subsequent experiments.

dCas9-GFP and sgRNA 3 expression vectors were transfected into HEK293, hTERT-RPE1, HFF1, TIG1, and MRC5 cells to confirm CRISPR imaging of α-satellite DNA in other human cell lines. The number of GFP domains in the nucleus was dependent on the cell types (Fig. 1c). In cell lines with 46 chromosomes and a normal chromosome number (hTERT-RPE1, HFF1, TIG1, and MRC5), > 50% of cells showed eight GFP domains in the nucleus (Fig. 1d). In contrast, cell lines with a relatively large number of chromosomes (HeLa and HEK293) showed > 10 GFP domains in the nucleus (Fig. 1d).

### Characterising α-satellite DNA domains detected using CRISPR imaging

**Figure 2:**
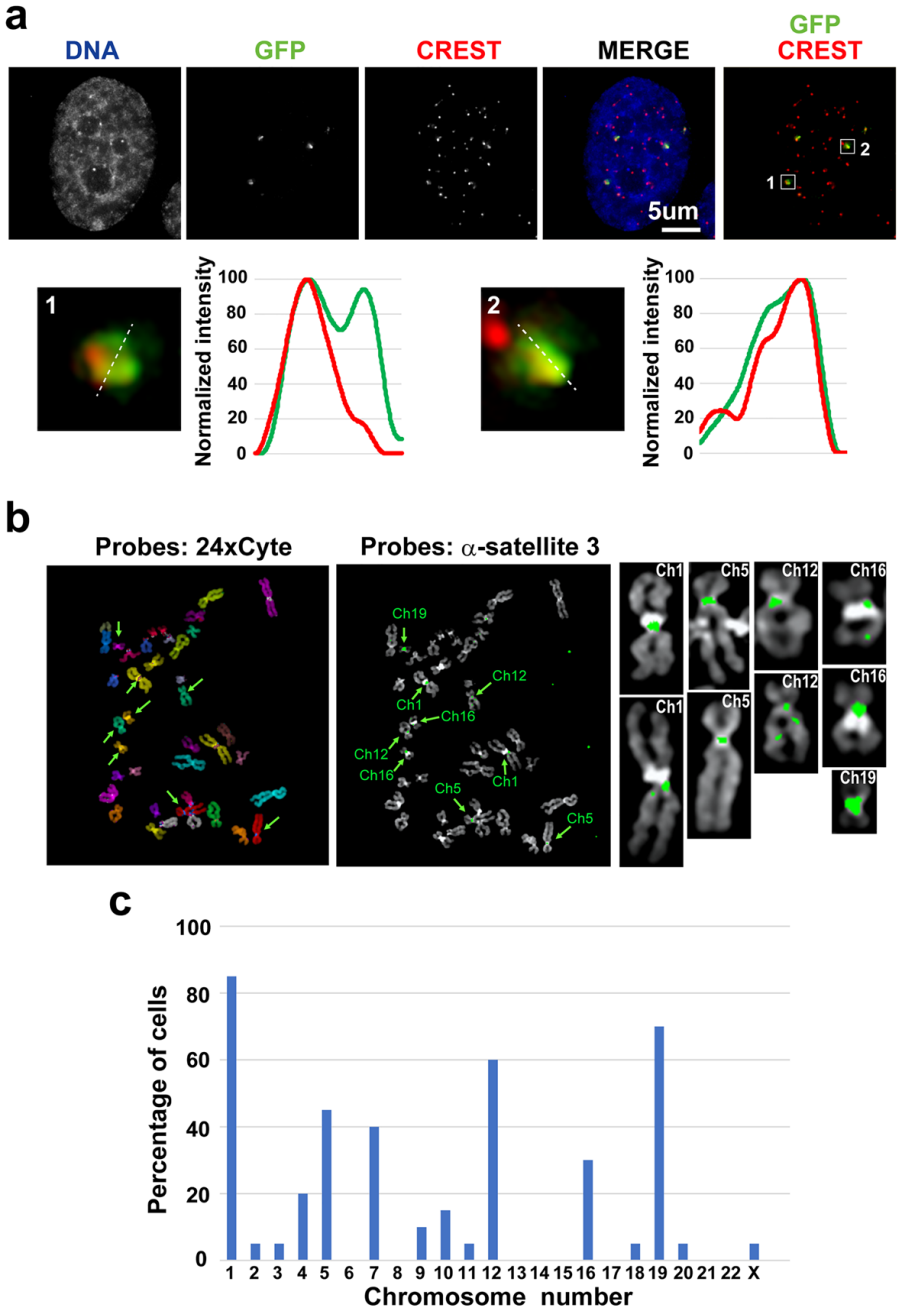
Immunostaining of centromeric proteins and multicolour fluorescence in situ hybridisation (FISH) using α-satellite DNA probes. (**a**) Centromere regions in hTERT-RPE1 cells were detected through CREST staining (red) and were combined with CRISPR imaging targeting α-satellite DNA (green). DNA was counterstained using Hoechst 33342 (blue). Two centromere regions enclosed by white boxes (1 and 2) are enlarged, and their line profiles, indicated by white dashed lines, are shown by graphs. (**b**) hTERT-RPE1 cell chromosomes were distinguished using 24 × Cyte probes, and individual chromosomes were detected using different colours (left). The hybridisation of DNA oligo probes with the targeting site of sgRNA 3 (α-satellite 3) showed green signals at the centromeres (right). Green arrows indicate chromosomes with signals of the α-satellite 3 probe, and enlarged chromosome images are also presented. (**c**) Percentage of cells showing α-satellite DNA signals on chromosomes 1–22 and X. Twenty cells were analysed.

Centromeric proteins were detected through immunostaining using anti-centromere antibody (CREST) staining to confirm whether GFP domains detected using CRISPR imaging targeting α-satellite DNA are centromeric regions. CREST staining detected centromeric regions in the nucleus, and GFP domains overlapped with CREST signals (Fig. 2a). However, the line profiles demonstrated that the signal intensity peaks of GFP and CREST differed, and the signal distribution of GFP was broader than that of CREST. Therefore, GFP domains detected using CRISPR imaging targeting α-satellite DNA enclosed the centromere region with different binding properties of centromeric proteins. The number of GFP domains was much less than that of the CREST foci in the nucleus, implying the preference for CRISPR imaging for chromosomes.

Individual chromosomes were recognized using multicolour FISH to identify chromosomes detected using CRISPR imaging targeting α-satellite DNA. sgRNA 3-targeting sites were simultaneously detected using Alexa 488-conjugated oligo DNA probes in hTERT-RPE1 cells. Although not all chromosomes showed signals for α-satellite DNA, signals were detected at the centromere on some chromosomes (Fig. 2b). Over 60% of cells showed signals for α-satellite DNA on chromosomes 1, 12, and 19 (Fig. 2c). Additionally, approximately 30–40% cells showed signals on chromosomes 5, 7, and 16 (Fig. 2c). However, signals on other chromosomes were rarely observed. Therefore, targeting α-satellite DNA using sgRNA 3 preferred the centromere region depending on the chromosome number.

### α-satellite DNA dynamics during cellular senescence

**Figure 3:**
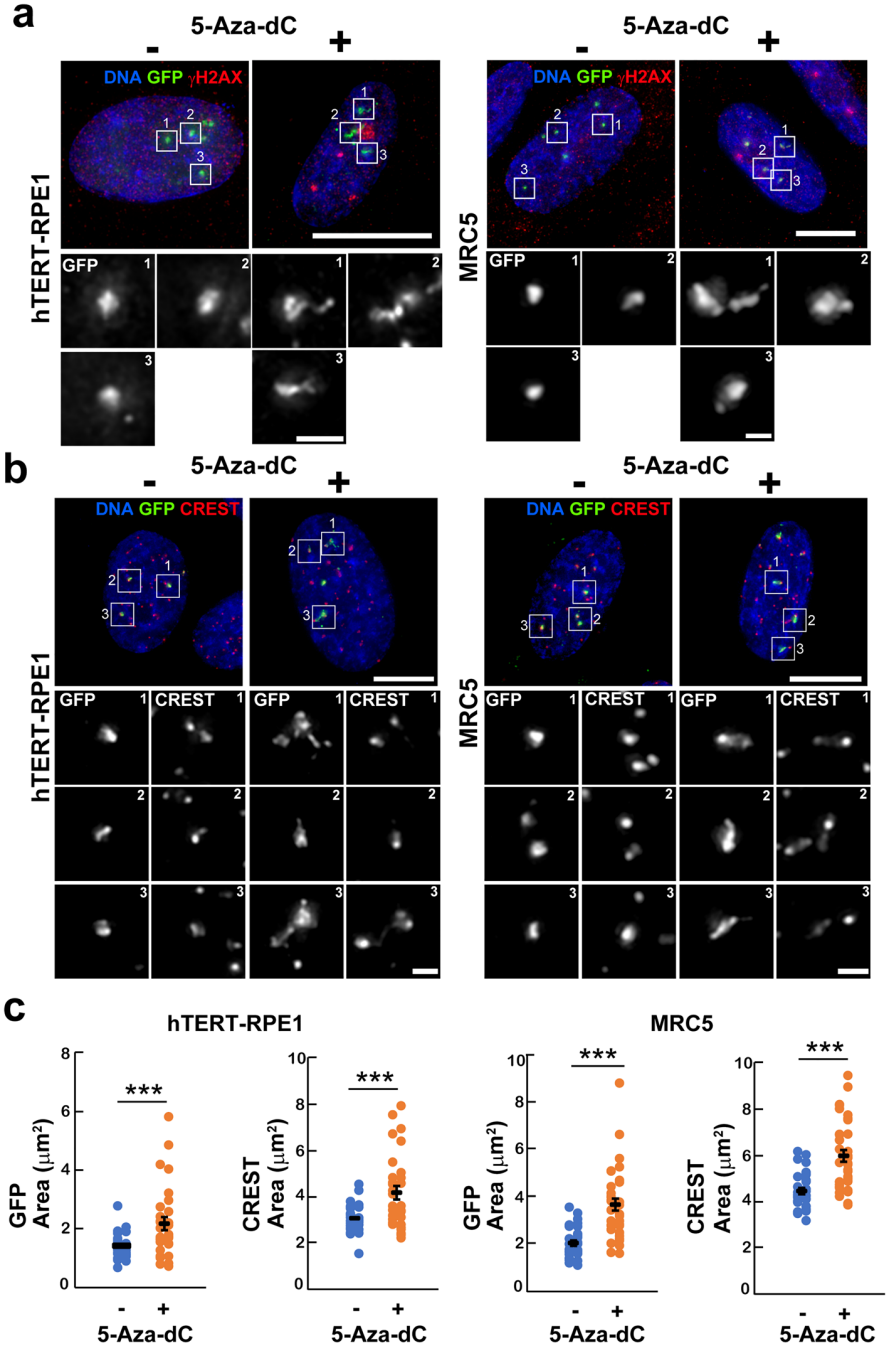
Changes in centromere organisation during cellular senescence. (**a**) Cellular senescence induction using 5′-aza-2′-deoxycytidine (5-Aza-dC) was confirmed by detecting γH2AX signals (red) in hTERT-RPE1 and MRC5 cells. α-satellite DNA signals detected by CRISPR imaging (green) showed expansion or elongation of the centromere regions. The centromere regions enclosed by white boxes are enlarged. DNA was counterstained using Hoechst 33342 (blue). Scale bars indicate 10 μm (upper panels) and 1 μm (bottom panels). (**b**) The GFP domain areas showing α-satellite DNA (green) and centromere protein domains detected using CREST antibody (red) were compared between nontreated and 5-Aza-dC-treated cells. The centromere regions enclosed by white boxes were enlarged. DNA was counterstained using Hoechst 33342 (blue). Scale bars indicate 10 μm (upper panels) and 1 μm (bottom panels). (**c**) The total area of the GFP and CREST domains in the nucleus of a single cell is plotted. The mean values (black lines) indicate with standard error of the mean (SEM). Over 25 cells were analysed. The *p*-value was calculated using Student’s *t*-test; ***p < 0.05.

The changes in chromatin organization at the centromere region during cellular senescence has been reported; however, its dynamics is poorly understood. Therefore, we applied CRISPR imaging targeting α-satellite DNA to chemically induced senescent cells. hTERT-RPE1 and MRC5 cells were treated with 5′-aza-2′-deoxycytidine (5-Aza-dC). The treatment caused global cytosine demethylation by inhibiting DNA methyltransferase (DNMT) by forming an irreversible covalent bond between DNMT and DNA strands incorporated by 5-Aza-dC^35^, therefore, inducing cellular senescence^36,37^. Cellular senescence induction was confirmed by immunostaining for senescence-associated beta-galactosidase (SA-β-gal), a widely used cellular senescence biomarker, and γH2AX phosphorylated at the histone H2AX C-terminal serine-139 incorporated into damaged DNA during cellular senescence. The SA-β-gal and γH2AX positive cell percentages were increased after 5-Aza-dC treatment (Fig. S1). Notably, 5-Aza-dC-treated cells showed expanded or elongated GFP domains targeting α-satellite DNA (Fig. 3a). GFP domain disorder was accompanied by changes in centromere protein localisation detected through CREST staining (Fig. 3b). The area of GFP and CREST signals within the nucleus was significantly enlarged in 5-Aza-dC-treated cells (Fig. 3c). The change in the chromatin structure at the α-satellite DNA region was also observed using another sgRNA which can detect centromere regions (Fig. S2). These results indicate that CRISPR imaging can reflect the changes in chromatin structure at the α-satellite DNA region during cellular senescence.

**Figure 4:**
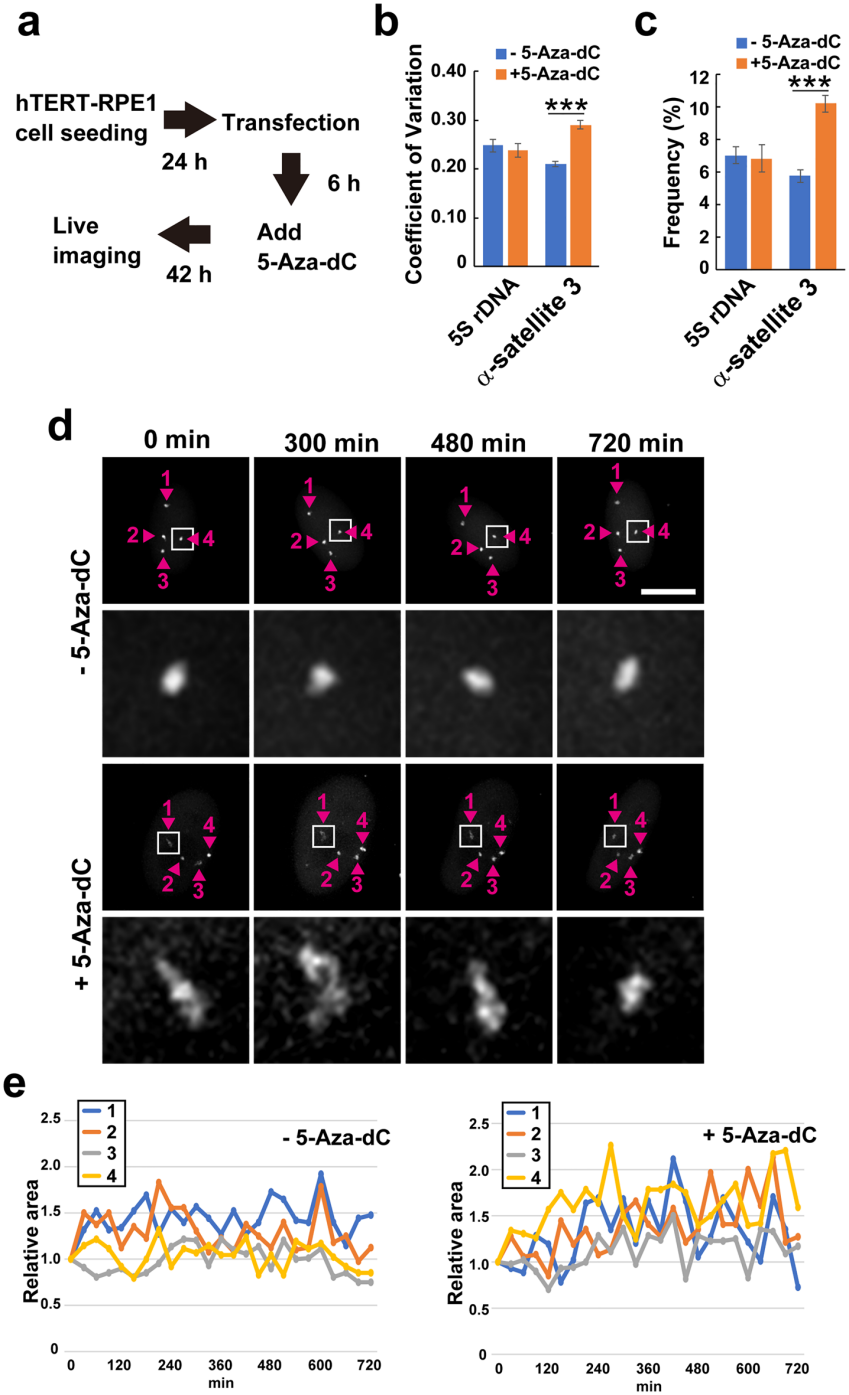
The dynamics of α-satellite DNA during cellular senescence. (**a**) Live imaging procedure of α-satellite DNA during cellular senescence. (**b**) Coefficient of variation of the GFP domain size in nontreated and 5-Aza-dC-treated cells. Over 75 GFP domains were analysed. The error bars show the SEM. The *p*-value was calculated using Student’s *t*-test; ***p < 0.05. (**c**) Comparison of GFP domain fluctuation. The frequency of the GFP domain area showing > 30% variation from the area at time 0 within 30 min during observation was calculated in nontreated and 5-Aza-dC-treated cells. The error bars show the SEM. The *p*-value was calculated using the Student’s *t*-test; ***p < 0.05. (**d**) Live observation of α-satellite DNA in nontreated and 5-Aza-dC-treated hTERT-RPE1 cells using CRISPR imaging. The GFP domain areas, indicated by arrowheads with numbers, were monitored during observation. The GFP domains enclosed by white boxes are enlarged. Scale bar: 10 μm. (**e**) The relative area to the size at 0 min for each GFP domain was monitored during live imaging. The numbers (1–4 for nontreated and 1–4 for 5-Aza-dC-treated cells) correspond to the numbers of GFP domains shown in (**d**).

hTERT-RPE1 cells were treated with 5-Aza-dC after transfection with dCas9-GFP and sgRNA expression vectors and were observed using a fluorescence microscope equipped with an incubator to monitor the changes in α-satellite DNA structure (Fig. 4a). The 5S ribosomal DNA (5S rDNA) region, located on chromosome 1 was also visualized using CRISPR imaging to compare the chromatin dynamics. Dynamic α-satellite DNA and 5S rDNA changes were observed in the living cells, and the coefficient of variation (CV) in the GFP domain size was calculated. The CV of α-satellite DNA in senescent cells [0.290 ± 0.009, mean ± standard error of mean (SEM)] was significantly larger than that of non-senescent cells (0.210 ± 0.006, mean ± SEM) (Fig. 4b). GFP area fluctuations of > 30% from the start of the observation were observed more frequently in the α-satellite DNA region of the senescent cells (10.17 ± 0.53%, mean ± SEM) compared to non-senescent cells (5.73 ± 0.40%, mean ± SEM) (Fig. 4c). In contrast to the α-satellite DNA, 5S rDNA did not show significant change in the chromatin dynamics in senescent cells (Fig. 4b,c). The typical images of the GFP domain dynamics with and without 5-Aza-dC treatment are shown in Fig. 4d and Supplementary Fig. 3. Untreated cells maintained the GFP domain size of α-satellite DNA (Fig. 4e, left). In contrast, treated cells showed fluctuations in the GFP domain size (Fig. 4e, right). Similar GFP domain size fluctuations in treated cells were also observed using another sgRNA targeting α-satellite DNA (Fig. S4). These results indicate that the fluctuation in chromatin structure at the α-satellite DNA increases during cellular senescence induced by 5-Aza-dC, and chromatin dynamics might be required for the centromere disorganisation during cellular senescence.

## Discussion

We developed a CRISPR imaging technique to observe α-satellite DNA dynamics in living cells and revealed the centromere-related disorders during SADS. The sgRNA targeting α-satellite DNA used in this study showed preference of chromosomes in CRISPR imaging as the number of GFP foci was less than that of the CREST signals. Detecting limited α-satellite DNA regions enabled us to efficiently track the dynamics of target regions by eliminating overlapping signals of other α-satellite DNA regions. The overlapping of several GFP domains was often observed using sgRNA targeting α-satellite DNA previously reported^33^, especially in the senescence-induced cells (Fig. S4c). Similar inconsistencies in the number of α-satellite DNA domains detected using CRISPR imaging and centromeres detected through other procedures such as CREST staining have been reported^32,33,38^. These inconsistencies might result from insufficient labelling efficiency or competition between the two methods.

The number of detected centromere regions differed among the cell lines. The difference in the number of GFP domains among cell lines might reflect the number of these chromosomes in cells. HeLa cells have substantial aberrations in chromosomes, with approximately 70 chromosomes. Chromosomal aberrations might increase the α-satellite DNA region targeted by our sgRNA. In normal cell lines, eight α-satellite domains were often detected in the nucleus through CRISPR imaging using sgRNA 3. The number is less than that in previous studies using other sgRNAs^32,33,38^. The centromere detection efficiency using CRISPR imaging depends on the sgRNA structure and the number of perfect matches for sgRNA in the genome^32,33^. The human genome has divergent α-satellite monomer units, and the number of monomer units comprising different sequences is more than 1000^39^. Some α-satellite DNA is organized into higher-order repeats (HOR) consisting of different monomeric units^40^. The HOR arrays have been classified into suprachromosomal families (SF) based on the monomeric configurations^41^. Our CRISPR imaging using sgRNA 3 preferentially detected chromosomes 1, 5, 7, 12, 16, and 19, categorized as SF1^42^. Therefore, our sgRNA design might reflect the HOR structure to some extent. The sgRNA 3 sequence used was designed from a consensus α-satellite sequence directly determined from human genome DNA after restriction enzyme digestion generating α-satellite dimers comprising 169 bp and 171 bp monomer units^31^. This method eliminated α-satellite DNA without restriction enzyme sites, and the sequenced α-satellite DNA should be at a limited locus. In contrast, sgRNA 5, designed from previously reported CRISPR imaging^33^, is based on a consensus sequence determined by comparing over 130 α-satellite monomer sequences from at least 14 different chromosomes^40^. Therefore, sgRNA 5 will cover more α-satellite regions than sgRNA 3. Thus, the difference in the reference α-satellite sequences leads to the difference in the number of α-satellite domains through CRISPR imaging.

Chemically induced cellular senescence using 5-Aza-dC disordered the centromeric structure. Global DNA demethylation caused by 5-Aza-dC treatment induced SADS within three days, and cells showed cellular senescence phenotypes. During 5-Aza-dC-induced cellular senescence, a relatively high α-satellite DNA domain fluctuation was observed using live CRISPR imaging. In addition to SADS, chromatin reorganisation, such as global loss and focal gains of heterochromatin and senescence-associated heterochromatin foci (SAHF) formation, is also closely associated with cellular senescence^21,43^. The changes in chromatin structure have been revealed using microscopic observation and chromosome conformation capture techniques^26,44,45^. The high α-satellite DNA domain fluctuation during cellular senescence might reflect the conversion of the chromatin structure from rigid to flexible. The rigid chromatin structure caused a fixed nuclear architecture and defined gene expression patterns required for normal cell proliferation. In contrast, the flexible chromatin structure generates a plastic nuclear environment to reorganize the nuclear architecture for cellular senescence. Histone modifications and chromatin-associated proteins cause changes in the chromatin structure. For example, histone H3 trimethylated at Lys9 and high-mobility group protein A are recruited in the SAHF region and contribute to condensed chromatin organisation^46,47^. The nuclear lamina also regulates the chromatin structure by anchoring chromatins to the nuclear envelope^48^. The disorganisation of the centromere structure, including SADS, observed in fixed cells is considered to be within the nuclear reorganisation process for cellular senescence. Actually, SADS is thought to be an early event in cellular senescence^27^. The high fluctuation of α-satellite DNA observed in this study might facilitate SADS.

In conclusion, our CRISPR imaging system is beneficial for tracking the dynamics of α-satellite DNA associated with the centromere during cellular senescence. Combining CRISPR imaging with the visualising chromatin components, such as centromeric proteins and histone modification, will contribute to understanding the precise reorganisation mechanism of the nuclear architecture causing cellular senescence.

## Methods

### Plasmid construction

For CRISPR imaging, an sgRNA expression vector, pLH-spsgRNA 2 plasmid (Addgene, Watertown, MA, USA), was amplified, and the oligonucleotides targeting α-satellite DNA repeats (sgRNA1-5)^33,49^ and 5S rDNA^34^ were inserted into the *Bbs*I site as previously described^16^. The sgRNA sequences were as follows:

sgRNA1: 5′-CTTTTTGTGGAATTTGCAAG-3′

sgRNA2: 5′-GACTTGAAACACTCTTTTTG-3′

sgRNA3: 5′-TGGAGATTTCAGCCGCTTTG-3′

sgRNA4: 5′-TTCAGCCGCTTTGAGGTCAAT-3′

sgRNA5: 5′- GAATCTCCAAGTGGATATT-3′

5S rDNA: 5′- GGCCTGGTTAGTACTTGGAT-3′

The pHAGE-TO-dCas9-3 × GFP plasmid (Addgene) was used to express dCas9-GFP.

### Cell culture and transfection

Human cell lines, including HEK293 (embryonic kidney cells, Tet-ON 3G cell line, Clontech, Mountain View, CA, USA), HeLa (cervical cancer cells, Tet-ON 3G cell line, Clontech), HFF1 (foreskin fibroblast cells, American Type Culture Collection (ATCC, Manassas, VA, USA)), hTERT-RPE1 (hTERT-immortalized retinal pigment epithelial cells, ATCC), TIG1 (lung fibroblast cells, Japanese Collection of Research Bioresources (JCRB, Osaka, Japan) cell bank), and MRC5 (lung fibroblast cells, JCRB cell bank), were maintained in Dulbecco’s Modified Eagle Medium (DMEM, Gibco, Waltham, MA, USA) supplemented with 10% foetal bovine serum (FBS, Gibco) in a humidified incubator with 5% CO_2_ at 37 ℃. For CRISPR imaging, 1 × 10^5^ cells were cultured on poly-L-lysine-coated coverslips in a 24-well plate to observe fixed cells or on a glass bottom 24-well plate for live cell imaging for 24 h. Cells were transfected with 750 ng sgRNA and 150 ng dCas9-GFP expression vectors using Lipofectamine 3000 (Invitrogen, Waltham, MA, USA). Cells were used for imaging experiments after 48 h.

Cells were treated with 20 μM 5-Aza-dC (Sigma-Aldrich, St. Louis, MI, USA) for 72 h to induce cellular senescence, and the medium was changed daily.

### Antibodies

The following primary antibodies were used for immunostaining: Rabbit polyclonal anti-γH2AX (1:200; Cell Signaling Technology, Danvers, MA, USA), human nuclear ANA-centromere autoantibody (CREST) antiserum (1:1000; Cortex Biochem, San Leandro, CA, USA), and chicken polyclonal anti-GFP (1:1000; Abcam, Cambridge, United Kingdom).

### Immunostaining and fluorescence microscopy

Human cells cultured on poly-L-lysine-coated coverslips were washed with phosphate-buffered saline (PBS; 137 mM NaCl, 2.7 mM KCl, 10 mM Na_2_HPO_4_, and 2 mM KH_2_PO_4_, pH 7.4) and fixed with 2% formaldehyde in PBS for 15 min at room temperature. Fixed cells were permeabilized with 0.2% Triton X-100 in PBS for 5 min at room temperature. Cells were blocked using 1% bovine serum albumin in PBS for 30 min at room temperature. Cells were then incubated with primary antibodies at room temperature for 1 h and then with secondary antibodies at room temperature for 1 h. DNA was counterstained using Hoechst 33342. SA-β-gal was detected using a cellular senescence detection kit (Dojindo, Kumamoto, Japan) before fixation to detect cellular senescence. Coverslips containing cells were mounted onto glass slides using VECTASHIELD mounting medium (Vector Laboratories, Newark, CA, USA) and visualized using fluorescence microscopy, Delta Vision (Applied Precision, Issaquah, WA, USA) using a 1.4 NA PlanApo 100 × oil immersion objective (Olympus, Tokyo, Japan). The z-stacking distance was set at 0.2 µm. Raw 3D images were deconvoluted using constrained iterative deconvolution and converted into 2D images with maximum projection using softWoRx (Applied Precision). Images were converted into 8-bit grayscale and underwent background subtraction. Image processing and intensity measurement was done using ImageJ 1.53t software (National Institute of Health, Bethesda, MD, USA).

### Live cell imaging

hTERT-RPE1 cells were transfected with sgRNA and dCas9-GFP expression vectors as described above. After 6 h, cells were treated with 20 μM 5-Aza-dC for 42 h to induce cellular senescence. Before observation, DNA was counterstained with Hoechst 33342 for 15 min, followed by replacing the medium with phenol red-free DMEM (Gibco) containing 10% FBS, 4 mM L-glutamine, and 20 mM HEPES. 5-Aza-dC was added during observation for senescent cells. The time-lapse observation was conducted using Delta Vision (Applied Precision) equipped with a CO_2_ chamber set at 37 ℃. Oil immersion objective lens (60×, 1.42 NA PlanApoN) was used to obtain the z-stack images with a 0.5-µm distance. The z-stack images were captured every 30 min. The images were processed as described above. GFP domain areas were analysed using ImageJ 1.53t software, and the CV and relative area to the area at 0 min were calculated.

### Multicolour FISH

To obtain mitotic cells, 40% confluent hTERT-RPE1 cells were treated with 0.1 µg/ml colcemid for 12 h. Mitotic shake-off cells were washed with PBS, treated with a hypotonic solution (75 mM KCl) for 10 min at room temperature, and fixed with Carnoy’s solution (methanol:acetic acid, 3:1). Fixed cells were analysed using multicolour FISH (Trans Chromomics, Tottori, Japan) using human chromosome painting probes (24 × Cyte, MetaSystems, Medford, MA, USA) and an α-satellite DNA probe (5′-TGGAGATTTCAGCCGCTTTG-3′) labelled with Alexa 488 at its 5′ end.

### Supplementary Information

Supplementary Figures.

## Data Availability

The datasets generated or analysed during this study are available from the corresponding author upon reasonable request.
