# Supplementary material for: CRISPR imaging reveals chromatin fluctuation at the centromere region related to cellular senescence
Source: Sci Rep. 2023 Sep 5;13:14609. doi: 10.1038/s41598-023-41770-6 (PMC10480159; doi:10.1038/s41598-023-41770-6)
Supplement: Supplementary file 1 — Supplementary Figures. [file 41598_2023_41770_MOESM1_ESM.pdf]

Supplementary Information

CRISPR imaging reveals chromatin fluctuation at the centromere region related to cellular senescence

Hideaki Takata[1*], Yumena Masuda[2] & Nobuko Ohmido[2]

[1]Biomedical Research Institute, National Institute of Advanced Industrial Science and Technology (AIST), Ikeda, 563-8577, Osaka, Japan

[2]Graduate School of Human Development and Environment, Kobe University, Nada-ku, 657-8501 Kobe, Japan

*Corresponding author

E-mail: h.takata@aist.go.jp

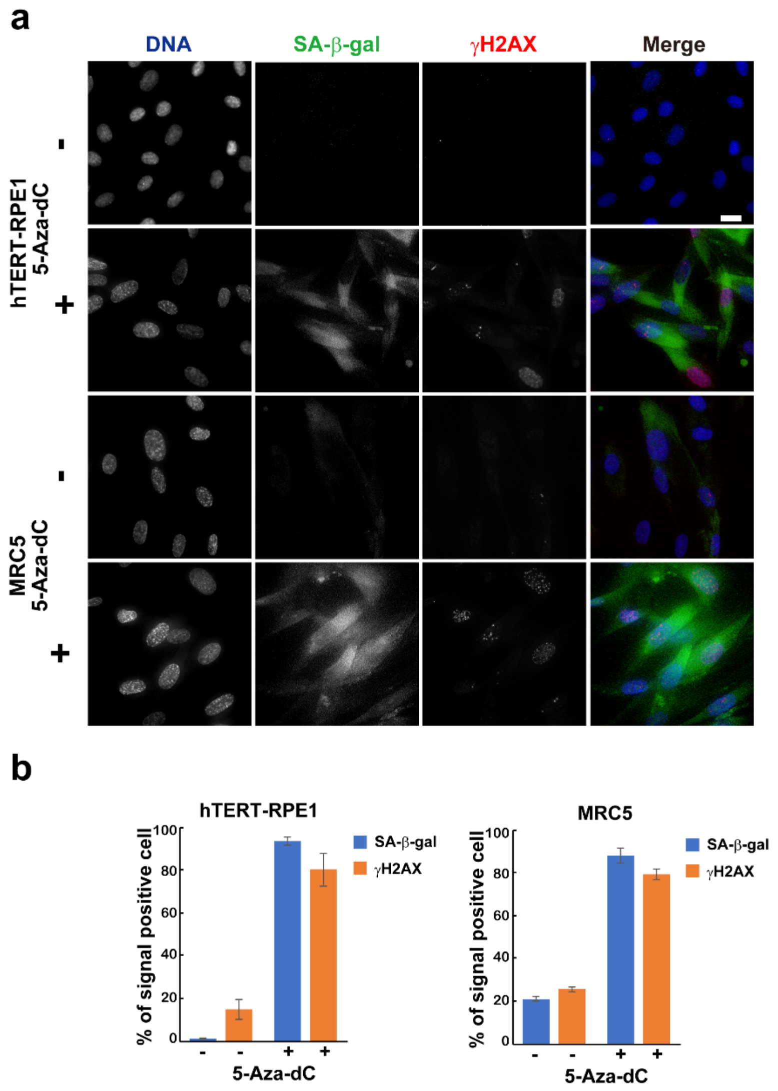

Supplementary Figure 1. Confirmation of cellular senescence induced by 5-Aza-dC

(a) The induction of cellular senescence by 5-Aza-dC was confirmed by detecting senescence-associated beta-galactosidase (SA-β-gal; green) and γH2AX (red) in hTERT-RPE1 and MRC5 cells. DNA was counterstained using Hoechst 33342 (blue). Scale bar indicates 20 μm. (b) The percentage of SA-β-gal and γH2AX positive cells in nontreated and 5-Aza-dC-treated cells. The error bars show the SEM. *n*=3 for both cell lines.

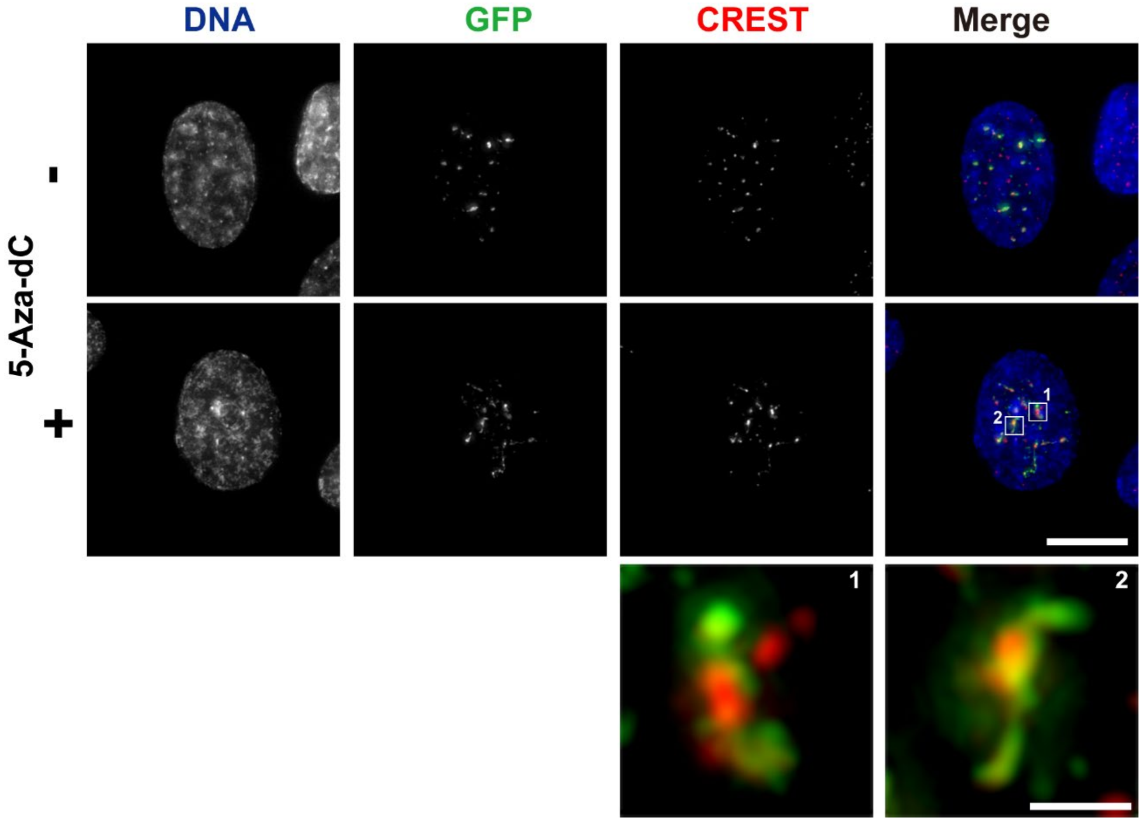

Supplementary Figure 2. Detection of α-satellite DNA using sgRNA 5

The signals of α-satellite DNA detected by CRISPR imaging (green) using sgRNA 5 in hTERT-RPE1 cells showed the increase in the number of GFP domains in a nucleus compared with using sgRNA 3. Centromere protein domains were detected by CREST antibody (red). The centromere regions enclosed by white boxes are enlarged. DNA was counterstained using Hoechst 33342 (blue). The expansion or elongation of the centromere regions were observed in 5-Aza-dC treated cells similar to CRISPR imaging using sgRNA 3. Scale bars indicate 10 μm (upper panel) and 1 μm (bottom panel).

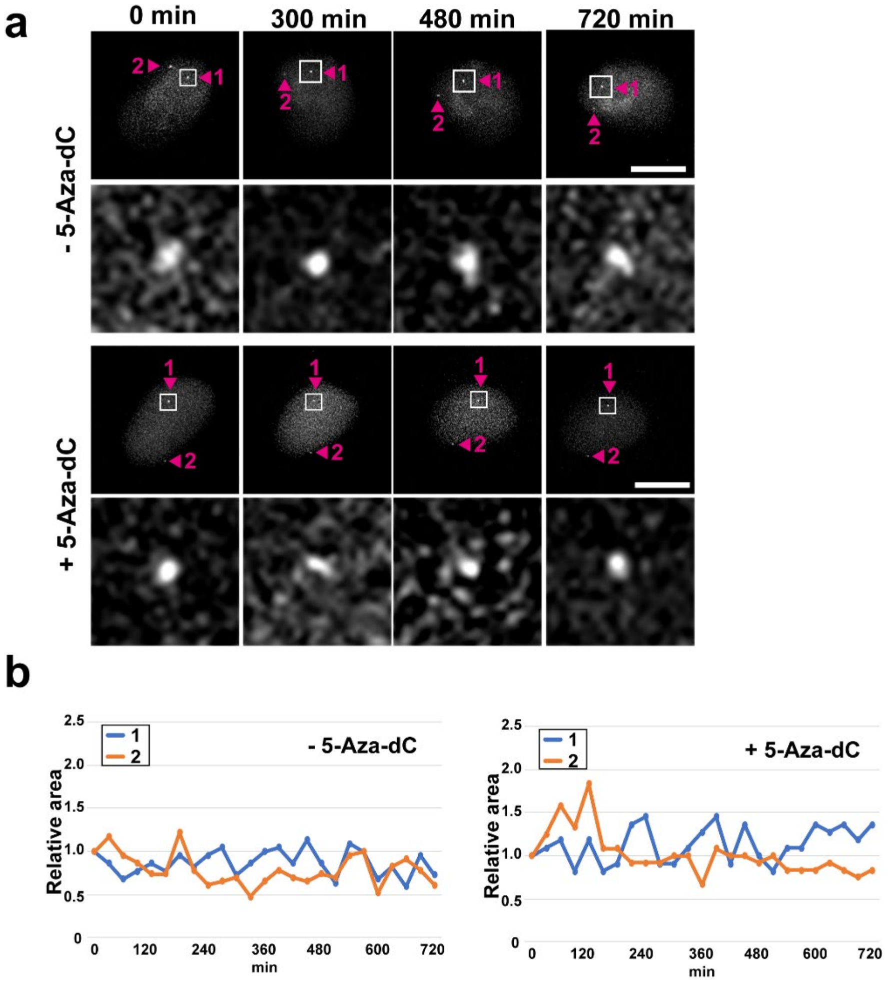

Supplementary Figure 3. The dynamics of 5S rDNA during cellular senescence

(a) Live observation of 5S rDNA in nontreated and 5-Aza-dC-treated hTERT-RPE1 cells using CRISPR imaging. The GFP domain areas, indicated by arrowheads with numbers, were monitored during observation. The GFP domains enclosed by white boxes are enlarged. Scale bar indicates 10 μm. (b) The relative area to the size at 0 min for each GFP domain was monitored during live imaging. The numbers correspond to the numbers of GFP domains shown in (b).

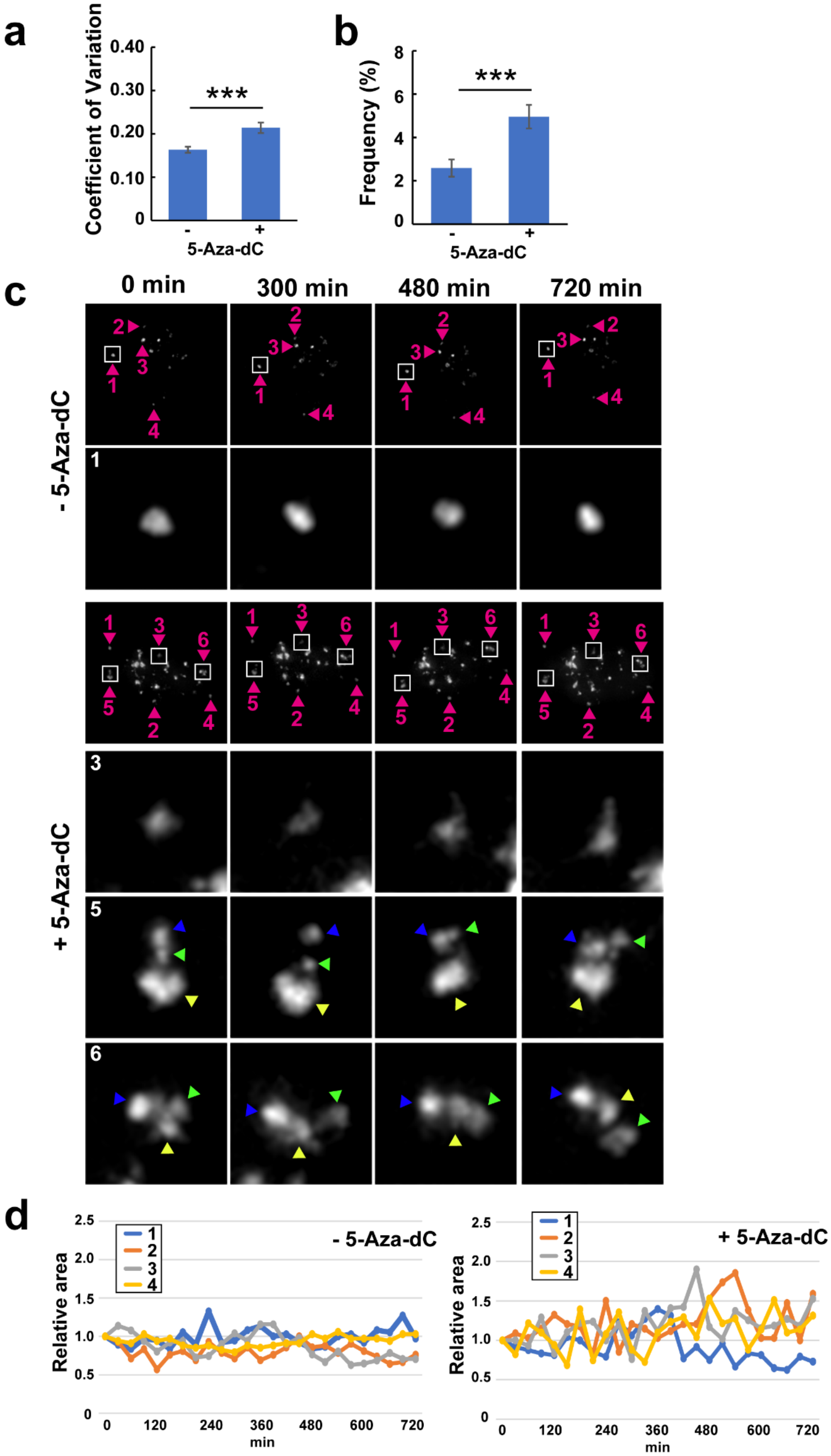
a
Coefficient of Variation
0.40
0.30
0.20
0.10
0
***
-
+
5-Aza-dC
b
Frequency (%)
8
6
4
2
0
***
-
+
5-Aza-dC
c
0 min
300 min
480 min
720 min
- 5-Aza-dC
+ 5-Aza-dC
d
Relative area
2.5
2.0
1.5
1.0
0.5
0
0
120
240
360
480
600
720
min
- 5-Aza-dC
+ 5-Aza-dC
1
2
3
4

Supplementary Figure 4. The dynamics of α-satellite DNA detected using sgRNA 5 during cellular senescence

(a) Coefficient of variation of the GFP domain size in nontreated and 5-Aza-dC-treated cells. Over 20 GFP domains were analysed. The error bars show the SEM. The p-value was calculated using Student's t-test; ***$p<0.05$. (b) Comparison of GFP domain fluctuation. The frequency of the GFP domain area showing >30% variation from the area at time 0 within 30 min during observation was calculated in nontreated and 5-Aza-dC-treated cells. The error bars show the SEM. The p-value was calculated using the Student's t-test; ***$p<0.05$. (c) Live observation of α-satellite DNA in nontreated and 5-Aza-dC-treated hTERT-RPE1 cells by CRISPR imaging. Live observation of α-satellite DNA in nontreated and 5-Aza-dC-treated hTERT-RPE1 cells using CRISPR imaging. The GFP domains enclosed by white boxes are enlarged. In the enlarged imagens, blue, green and yellow arrowheads indicate an individual GFP domain. Scale bar indicates 10 μm. (d) The relative area to the size at 0 min for each GFP domain was monitored during live imaging. The numbers (1–4 for nontreated and 1–4 for 5-Aza-dC-treated cells) correspond to the numbers of GFP domains shown in (c).
